# Abdominal Cystic Echinococcosis Treated with Albendazole. A Pediatric Cohort Study

**DOI:** 10.1371/journal.pone.0160472

**Published:** 2016-09-02

**Authors:** Samanta Moroni, Guillermo Moscatelli, Facundo García Bournissen, Nicolás González, Griselda Ballering, Héctor Freilij, Fabián Salgueiro, Jaime Altcheh

**Affiliations:** 1 Parasitology and Chagas Service, Buenos Aires Children Hospital Ricardo Gutiérrez, Capital Federal, Buenos Aires, Argentina; 2 Department of Surgery, Buenos Aires Children Hospital Ricardo Gutiérrez, Capital Federal, Buenos Aires, Argentina; Centers for Disease Control and Prevention, UNITED STATES

## Abstract

**Introduction:**

Cystic echinococcosis is endemic in Argentina. The standard pharmacological treatment for the disease is albendazole, but surgery is a common alternative. Even though primary infection occurs mainly in the pediatric population, the optimal therapeutic option in pediatrics is not clearly defined and few pediatric cohorts with cystic echinococcosis treated with albendazole have been described to date.

**Objective:**

To describe therapeutic response to albendazole in a cohort of pediatric patients with abdominal cystic echinococcosis.

**Population and Methods:**

Patients (0–18 years old) with abdominal cystic echinococcosis who were treated with albendazole between January 1998 and August 2013. Diagnosis of abdominal cystic echinococcosis was made by ultrasound. All patients received albendazole, 10–15 mg/kg/day. Epidemiological data, symptoms, number, location and outcome of the cysts, serology and treatment received were analyzed. The parameter used to assess treatment response was cyst changes evaluated by ultrasound follow up using the WHO-IWGE classification.

**Results:**

A total of 28 patients (with 46 abdominal cysts) were included in the cohort. Mean age at enrolment was 9.4 years and mean duration of follow-up, 23.8 months. All patients resided in rural areas and had had contact with dogs. The asymptomatic form of the disease was the most common presentation. All patients received albendazole (mean duration: 142.5 days), with low incidence of adverse events. Albendazole had a positive effect on most of the cysts. Surgery was performed in 13 patients.

**Conclusion:**

Treatment with albendazole for uncomplicated cystic echinococcosis cysts is safe and effective, and can potentially reduce the need for surgical intervention.

## Introduction

Cystic echinococcosis, also called Hydatid disease in the past, is a parasitic disease caused by the cestode *Echinococcus granulosus*. It is a zoonosis of worldwide distribution, with a major impact on public health. Children are at increased risk as they have closer contact with the definitive host (dog). The liver is the organ most frequently affected, and the most common presentation is asymptomatic [1]

Cystic echinococcosis is endemic in Argentina, particularly in rural areas as the infection occurs through dogs with no veterinarian control or deworming treatment.

Several recent studies have provided support for medical treatment of cystic echinococcosis with antiparasitic drugs such as the benzimidazoles albendazole (ABZ) and mebendazole (MBZ) [1, 2]. However, first line treatment with these drugs is not accepted by all experts, and there is limited evidence on the effectiveness and safety of ABZ in children, the most commonly used drug for cystic echinococcosis in this population [1].

The aim of this study was to describe a cohort of pediatric patients with abdominal cystic echinococcosis treated with ABZ.

## Population and Methods

We describe a retrospective cohort of pediatric patients, diagnosed with abdominal cystic echinococcosis treated with ABZ between January 1998 and August 2013 at the Parasitology and Chagas Service, Buenos Aires Children's Hospital “Ricardo Gutierrez”. Patients were included in the cohort if they were less than 18 years of age at the time of diagnosis, and had not received previous parasiticide medication. Demographic, epidemiological, clinical diagnosis, treatment, number, location and development of cysts and serological data were analyzed.

Cysts detected by ultrasound, classified according to the WHO-IWGE classification (Table 1) and visualization of parasitic elements removed after surgery were used as diagnostic criteria [3, 4]. Ultrasound (US) was performed by non-blinded pediatrics radiologists with plenty expertise in pediatric scan.

**Table 1:** WHO-IWGEs classification (modified from Brunetti E, Kern P, Vuitton DA, and Writing Panel for the WHO-IWGE (2010). Expert consensus for the diagnosis and treatment of cystic and alveolar echinococcosis in humans. Acta Tropica 114: 1–16).

Type of cyst	Characteristics
CL	Univesicular cyst lesion with anechoic content, normally round with no cyst wall visible. Normally these are non-parasitic lesions.
CE1 Active	Univesicular simple cyst that may exhibit fine echoes which is often called hydatid sand (“snow flake sign”). Cyst wall is visible.
CE2 Active	Multivesicular, multiseptated cysts in which the daughter vesicles may partly or completely fill the univesicular mother cyst. Cyst septations may produce “wheel-like” structures or the contained daughter vesicles may produce a “rosette-like” or “honeycomb-like” structure. Cyst wall is normally visible
CE3 Transitional	This is a transitional stage, where cysts are starting to degenerate. Cysts are less round due to decrease of intra-cystic fluid pressure. There are two types of CE3 cysts: CE3a, anechoic content showing in US examinations detachment and rupture of membranes (“water-lily sign”) and CE3b which is a univesicular cyst that may contain daughter vesicles and echoic areas appearing at US as a “complex mass”.
CE4 Inactive	Heterogenous hypoechoic or dyshomogeneous degenerative contents with no doughter vesicles. It may show a “ball of wool” sign, indicative of degenerating membranes
CE5 Inactive	Cysts with a calcified wall which is arch shaped, producing a cone shaped shadow. Calcification could be partial to complete

Specific serology was performed by indirect hemagglutination (IHA) (Hidatest, Lemos SRL Laboratory, Argentina). Results greater than or equal to 1/16 dilutions (DILS) were considered positive [5].

ABZ 10–15 mg/kg/day was administered orally in two doses per day with meals. Duration of treatment, dose of ABZ and adverse drug reactions (ADRs) were recorded. ABZ treatment length was decided according to the changes found on the US scan performed on the patients. Signs and symptoms suggesting ADRs were specifically inquired for and recorded during each visit. ADRs were defined according and assessed to World Health Organization definitions [6]. Causality assessment was performed using standardized criteria, [7] including the Naranjo score [8]. Additional clinical and laboratory evaluations were conducted during any unscheduled visit, and patients were advised to return on any day during the follow-up period if ADRs occurred. All concomitant medications were recorded. Physical examination and laboratory tests (complete blood counts, hepatic and renal function) were done every 15 days during treatment in order to detect ADRs.

Ultrasound studies (US) and IHA were performed on a monthly basis during treatment, and every 3–6 months afterwards. Evolution of cysts was assessed by monitoring US changes, as per WHO-IWGE´s classification [8]. To analyze treatment effect only those patients that had received at least one month of treatment with ABZ were taken into account. We included patients with concomitant lung cyst, but these lung cysts were not included in the statistical analysis. During and after treatment, disappearance or calcification of cysts was considered “cure”; appearance of new cysts was considered reinfection or failure of treatment; no modification in US was considered “deterioration” and modification in US was deemed “improvement” or “regression” (progression from one stage of WHO classification to the next). Improvement was the criteria used to stop treatment. Follow-up was performed for long periods of time in order to detect the appearance of new cysts.

Annual US was conducted to evaluate relapse and / or reinfection during follow-up.

All patients were studied with chest X-rays to evaluate presence of cysts in other locations. Abdominal computed tomography (CT) was performed when appropriate, mostly to decide surgical procedures or to confirm cyst location.

Family screening (serology, chest X-rays and abdominal US) was conducted for all cases in order to detect new cysts among the patient’s family.

In the cases requiring surgery, the surgical technique used was open surgery or video assisted surgery with complete removal of the proligerous membrane after sterilization with hypertonic saline solution.

Dogs were considered to have adequate veterinarian control if they had an annual visit to the veterinarian and deworming treatment every 4 months.

Student’s t and Mann Whitney tests were used to compare continuous and categorical variables, respectively. Continuous variables are presented as means with 95% confidence intervals and medians and interquartile ranges. Statistical analyses were performed in R (R Development Core Team (2011). R: A language and environment for statistical computing. R Foundation for Statistical Computing, Vienna, Austria. ISBN 3-900051-07-0, URL http://www.R-project.org/).

## Ethics Statement

The Children’s Hospital “Ricardo Gutierrez” Ethics Committee and Review Board approved this research.

Written or oral informed consent was not obtained from all participants due to the characteristics of the study (retrospective cohort) and because in most cases the study population live in rural areas were phone contact was difficult. We explained this problem to the ethics committee and they agreed to approve the research despite this issue.

## Results

**Fig 1:**
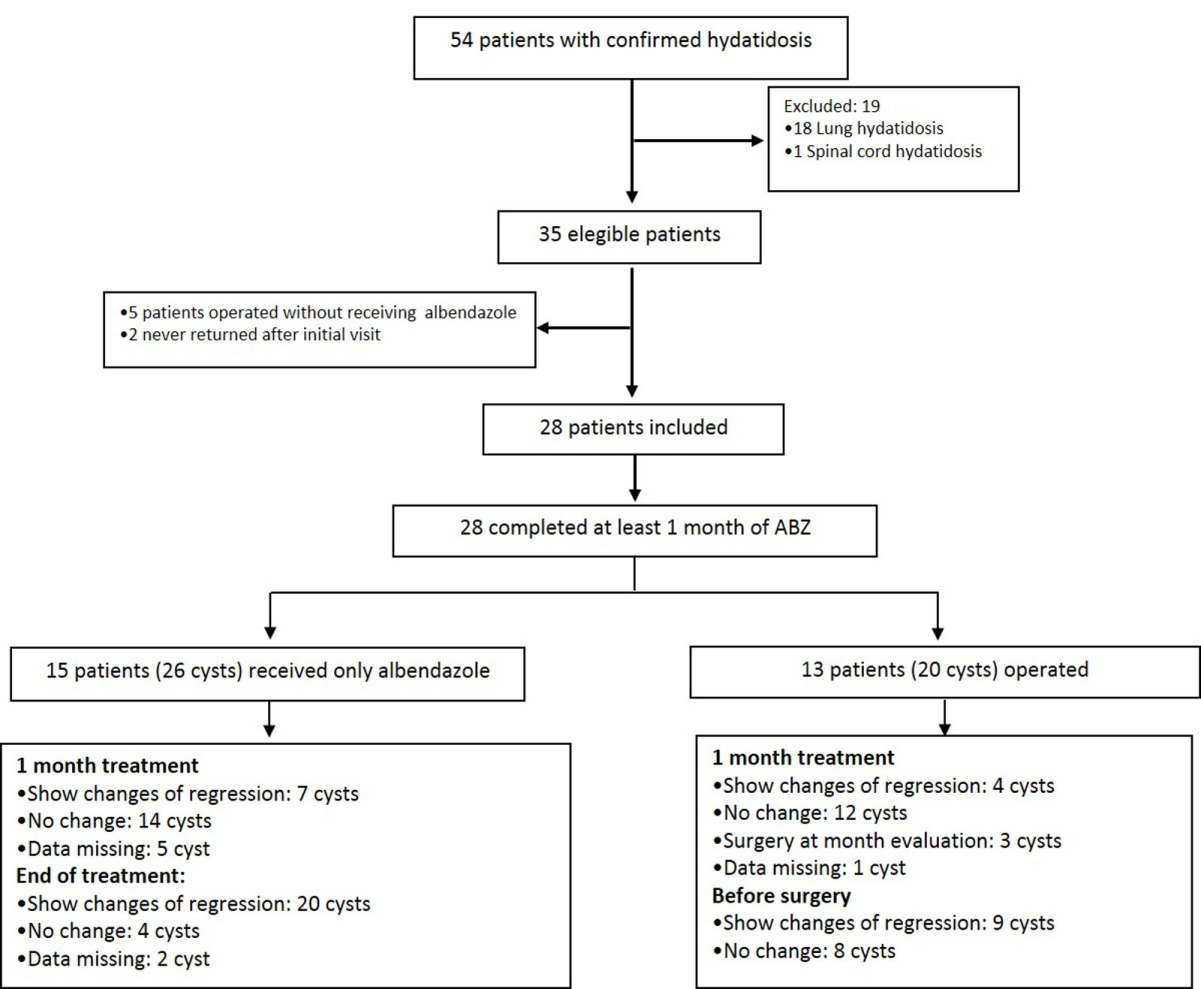
Flowchart of the patient inclusion process.

Out of the 54 patients with confirmed diagnosis of cystic echinococcosis followed in our service during the study period, 28 (51%) patients had abdominal cysts and fulfilled inclusion criteria for the cohort (Fig 1, Flowchart). All patients had had contact with dogs and a history of residence in rural areas mostly in Argentina (75%), or Bolivia (14.5%) (Table 2).

**Table 2:** Demographic and clinical data.

	Patients included in the data analysis n: 28	
Gender	• 12 male /16 female	• 43% Male
Age at diagnosis	• Mean 9.4 years (3–17 years) SD 4.19	
Country where infection acquired	• Argentina • Bolivia • Brasil • Paraguay • Perú	• 21 (75%) • 4 (14.5%) • 1 (3.5%) • 1 (3.5%) • 1 (3.5%)
Contact with dogs		100%
Dogs without veterinary control or deworming treatment		70%
Clinical presentation	• Asymptomatic • Abdominal pain • Pain and vomiting • Pain and fever • Only pain • Choledochal syndrome and abdominal pain • Palpable tumor • Cough and hemoptysis • Anaphylactic reaction • Pneumonia and vomica	• 15 (54%) • 4 (14%) • 2 (50%) • 1 (25%) • 1 (25%) • 2 (7%) • 3 (10%) • 2 (6%) • 1 (3%) • 1 (6%)
Serology at diagnosis	• Positive (> = 1/16) • Negative • No data	• 15 (53%) • 12 (43%) • 1 (4%)

The 28 patients had 46 abdominal cysts (42 cysts located in the liver, 2 in the kidney, 1in the spleen and 1 in the mesentery). Eight patients also had concomitant lung cysts. These cysts were not taken into account in the analysis. Mean age at diagnosis was 9.4 years (range 3 to 17 years). The median follow-up was 23.8 months (range 1 to 116 months), with an interquartile range 5.85–33. The most common presentation was asymptomatic in 13 patients (46%). These diagnoses were mostly incidental US findings from US performed for nonspecific symptoms (such as dyspepsia or abdominal discomfort). These US were not performed thinking of abdominal cystic echinococcosis. The 15 symptomatic patients (54%) presented with clinical signs or symptoms reflecting mostly internal organ compression by the cysts (Table 2). The liver was the main organ involved, in 25 patients (91.3%), followed by kidney in 1, spleen in 1, and kidney and mesentery in 1 patient. Eighteen patients (64.3%) had single cysts and ten (35.7%) had more than one abdominal cyst.

A total of 15/28 patients (53%) had positive serology at diagnosis. The median value was 8 DILS (min. 0—max. 512) at diagnosis and 24 DILS (min. 0—max. 512) at the end of follow-up of. After two years of follow-up (mean 23 months), 2 out of the 15 patients with positive values​​ became negative (one of them received only ABZ and the other one underwent surgery). Finally, among the 13 patients with initial negative serology, 6 became positive after treatment. Five of them were operated, but received also ABZ and 1 received only ABZ.

Chest x-rays were obtained in 25 subjects, and found to be abnormal in 8 (28%) patients.

Abdominal CT was performed on 16 patients: in 2 patients to confirm the organ involved (spleen, kidney) and in 14 to better assess therapeutic response. The results of the CT confirm the liver involvement and help the surgeon to decide whether or not operate on the patients according to cysts size and compression of adjacent organs to the liver.

All patients received ABZ (10–15 mg/kg/day). Mean duration of drug treatment was 142.5 days (range 30–331 days). ADRs were observed in 7 (25%) patients: increased liver enzymes in 5 (71.4%), bloody diarrhea in 1 (14.2%) and dizziness in 1 patient (14.2%). None of the patients had to discontinue the medication for such events. Among patients with increased liver enzymes, the mean value of AST was 2.2 times higher than the baseline (range 1.3–3.8 times) and ALT was 1.9 times higher (range 1.4–2.6 times). Liver enzymes returned to normal during treatment with ABZ.

At diagnosis, 30 cysts (65%) corresponded to type CE1, 8 (17%) to type CE2, 6 (13%) to type CE3, 1 (2%) to type CE4 and 1 to CE5 (2%). After one month of treatment, there were fewer type CE1 cysts and an increase of the other type of cysts showing a progression towards regression. At the end of treatment, there was 1 (2%) type CE1 cyst, 12 (26%) type CE2, 9 (20%) CE3, 8 (17%) CE4 and 6 (13%) CE5 (Table 3).

**Table 3:** Evolution of cysts treated with albendazole.

	CE1 n (%)	CE2 n (%)	CE3 n (%)	CE4 n (%)	CE5 n(%)	Surgery	Data not available
Pretreatment	30 (65)	8 (17)	6 (13)	1 (2)	1 (2)	0 (0)	0 (0)
1 month treatment	12 (26)	17 (37)	5 (10)	1 (2)	1 (2)	3 (7)	7 (16)
End of treatment	1 (2)	12 (26)	9 (20)	8 (17)	6 (13)	8 (17)	2 (5)
End of follow up	0 (0)	10 (22)	7 (15)	6 (13)	7 (15)	9 (20)	4 (9)

Mean cyst size at the beginning of ABZ treatment was 63 mm (range 17 to 203 mm) and 41 mm at the end (range of 0 to 160 mm). The decrease in the size of the cysts after treatment was statistically significant (median difference -15 mm, 95% CI [-27 to -8 mm], P <0.001). (Paired t-test). Table 4.

**Table 4:** Cysts details and evolution during and after treatment. NA: Data not available.

CYST NUMBER	SIZE AT DIAGNOSIS (mm)	SIZE POSTTREATMENT (mm)	WHO CLASIFICATION. PRETREATMENT	WHO CLASIFICATION AT .1m TREATMENT	WHO CLASIFICATION POSTREATMENT	WHO CLASIFICATION AT END OF FOLLOW UP	SURGERY	DAYS OF TREATMENT	CYST LOCALIZATION	Time of follow up (months)
1	69	61	1	2	2	2	no	100	Liver	54
2	47	46	1	2	2	2	no	100	Liver	54
3	178	NA	1	NA	NA	NA	no	61	Liver	54
4	73	NA	1	NA	NA	NA	no	61	Liver	15
5	101	61	1	2	3	3	no	132	Liver	12
6	70	36	2	2	5	5	no	157	Liver	43
7	74	47	1	1	5	5	no	157	Liver	43
8	51	36	1	1	5	5	no	157	Liver	43
9	19	19	1	1	3	3	no	143	Liver	12
10	82	60	2	2	4	4	no	140	Liver	21
11	35	50	1	1	4	4	no	162	Liver	43
12	62	31	1	NA	5	5	no	179	Liver	1
13	42	23	1	NA	5	NA	no	179	Liver	21
14	43	34	1	NA	4	5	no	179	Liver	5
15	52	34	3	4	4	NA	no	179	Liver	5
16	30	32	2	2	4	4	no	179	Liver	19
17	40	44	4	NA	4	4	no	179	Liver	6
18	53	51	1	2	2	2	no	127	Liver	3
19	75	53	3	3	4	4	no	142	Liver	3
20	40	38	1	1	2	2	no	138	Liver	2
21	59	44	1	1	2	2	no	138	Liver	2
22	47	40	2	2	2	NA	no	135	Liver	10
23	35	25	1	2	3	3	no	189	Liver	11
24	80	50	1	2	2	2	no	177	Liver	11
25	50	42	3	3	3	3	no	177	Liver	30
26	57	58	3	3	3	3	no	177	Liver	30
27	49	13	2	2	NA	2	yes	147	Liver	4
28	73	NA	2	2	NA	NA	yes	147	Liver	4
29	50	NA	2	2	NA	NA	yes	147	Liver	1
30	65	66	3	3	4	4	Yes	262	Liver	1
31	34	NA	1	NA	3	3	yes	262	Liver	17
32	76	81	1	2	2	2	yes	202	Spleen	17
33	91	64	1	NA	3	3	yes	36	Liver	17
34	17	15	1	1	2	3	yes	213	Liver	19
35	52	55	1	1	1	NA	yes	331	Liver	116
36	46	45	1	1	2	NA	yes	331	Liver	116
37	42	41	2	NA	NA	NA	yes	331	Liver	116
38	45	0	5	5	5	NA	yes	172	Liver	52
39	58	0	1	NA	NA	NA	yes	30	Liver	52
40	82	0	1	1	3	NA	yes	185	Liver	26
41	203	160	3	3	3	3	yes	218	Liver	40
42	59	32	1	2	2	2	yes	58	Liver	73
43	62	59	1	2	2	2	yes	58	Kidney	23
44	112	0	1	1	NA	NA	yes	122	Mesentery	54
45	70	70	1	2	2	2	yes	297	Kidney	54
46	74	87,5	1	1	1	2	yes	297	Liver	54

All patients received at least one month of ABZ (Fig 1), 15 received only drug therapy with ABZ and 13 patients were operated. Mean duration of treatment in those patients who received only ABZ was 157 days (SD 45 days) and for those who were operated was 202 days (SD 95 days). The 15 patients who were treated only with ABZ had 26 hepatic cysts with no concomitant lung localization. After one month of ABZ, 7 cysts show changes of regression in WHO’s classification, (6 cyst CE1 change to CE2 and 1 CE3 change to CE4); 14 cysts showed no change (6 were CE1, 4 CE2, 3 CE3 and 1 CE4) and data was missing for 5 cysts. At the end of ABZ treatment 20/26 cysts show changes of regression WHO’s classification and 4 cysts show no change. Those last cysts were type 3 and 4. Data was missing for 2 cysts. Notably 8 of the 26 cysts were bigger than 7 centimeters. At the end of follow-up, all cyst progress to regression and particularly 3 cysts could not be observed by ultrasound.

One patient had a type CE5 cyst which would have been a candidate for the “watch and wait” modality but received ABZ for treatment of a lung cyst.

The evolution of the cysts operated (13 patients with 20 cysts) was as follows: after one month treatment with ABZ, 4 cysts CE1 change to CE2; 12 cysts show no change, data was missing for 1 and 3 patients were operated just before the evaluation at 30 days treatment. Before surgery was performed, 9 cysts show changes of improvement and 8 cysts show no change.

Surgical treatment was performed in 13/28 (46%) patients (20 abdominal cysts). Video laparoscopy was performed in 5 patients (38%). No patients operated by video laparoscopy required conversion to open surgery and there were no complications due to the procedure. The reason for surgery was atypical location in 3 patients (kidney, spleen, and mesentery); large cyst size in 2 patients; complication (vomica) of extrabdominal location (lung) in 4 patients, (surgeon’s decision to opportunistically operate the concomitant hepatic location); in 2 patients the surgeon decided to operate the abdominal cysts without formal indication; in 1 patient for cyst size and persistence of abdominal pain and in 1 patient due to rupture of a liver cyst. During surgery, cystic samples were taken for microscopy to confirm the diagnosis finding in 5 cases scolex and hydatid sand. After microscopic pathology evaluation, confirmation of the diagnosis was obtained in all operated patients.

Surgical complications observed were nosocomial pneumonia in one patient and bilioma in other patient.

## Discussion

In Argentina, cystic echinococcosis is widespread throughout the country, being more prevalent in the rural areas of the Central and Southern regions of the country. Epidemiological notifications by age group evidence that the disease is detected more often in older people (median age: 39 years), with a relatively small number of new pediatric cases diagnosed every year. This reflects a significant underreporting of this pathology, since the infection is commonly acquired early in life, followed by a silent evolution for years [4].

During the period studied (1998–2013), our Service, a pediatric referral center, treated 54 patients with confirmed diagnosis of abdominal cystic echinococcosis, most of whom were from Argentina’s Central (Buenos Aires 43.2%) and North (Santiago del Estero 33.3%) regions.

In recent years a paradigm shift in the treatment of cystic echinococcosis has taken place, from a predominantly surgical approach to a more comprehensive approach based on medical treatment. Four treatment options currently exist: surgery, PAIR (Puncture, aspiration, injection, and re-aspiration), drug treatment with ABZ, and watchful waiting [9, 10]. The choice and timing of these therapeutic options are very much dependent on cyst and patient characteristics, but also on medical choices that may not be completely evidence based.

The therapeutic efficacy of ABZ as a single intervention in uncomplicated cysts was demonstrated in studies by Gil Grande [2], Larrieu [11] and other authors, where prolonged treatment with this drug showed degenerative changes in the cysts (suggesting decrease in viability), making surgery unnecessary [2, 9, 10, 11].

No guidelines currently exist for treatment of abdominal cystic echinococcosis in children, recommendations being based mostly on expert opinion. Our treatment decisions followed WHO guidelines, and Brunetti et al, which suggest that the choice of treatment should be based on the stage of the cyst, its location and size, patient comorbidities and team experience in treating the condition [1, 12, 13]. Based on our observations, we believe that ABZ treatment should be stop when US shows changes of “improvement”.

Ultrasound is currently the mainstay of the diagnosis and for long-term follow-up, since it does not require the use of radiation, or general anesthesia, is easily available and of low cost. Although the best way to evaluate the cystic viability is by surgical removal of cysts and microscopic evaluation and subsequent inoculation on mice, from ultrasound changes proposed by WHO-IWGE and Gharby [14], we can establish the therapeutic efficacy of ABZ [15]. This diagnostic procedure is highly specific to show changes in cystic viability but of low sensitivity. Other methods have been proposed as the nuclear magnetic resonance spectroscopy to assess cyst metabolic profiles, but these procedures are costly and still not validated [16].

Most cysts found in our cohort were type CE1 (30/46 cysts), reflecting that most viable cysts in the pediatric population are uncomplicated, and have excellent response to medical treatment. In our cohort all this type of cyst progress to regression due to the ABZ treatment nevertheless the size or location of the cyst.

Previous published studies [11] rarely demonstrated cystic disappearance possibly because most patients underwent surgery or were not monitored long-term. Among our patients, cystic disappearance was observed in 3 patients by ultrasound, which may be related to longer periods of monitoring and treatment implemented in our cohort.

As described by Todorov [17], some cysts seemed to respond better than others, even within the same patient; that was the case in one patient who had 2 liver cysts, one (CE1 type) with an excellent response to ABZ and another (CE3 type) that required surgical removal. For this reason it is necessary to confirm cystic evolution by US before surgical treatment is indicated.

ABZ was well tolerated by most patients when administered with fatty foods. This drug and mebendazole are the only approved treatments for abdominal cystic echinococcosis. In our department, we use ABZ given its better and more predictable intestinal absorption and low rate of adverse events [2, 18, 19, 20]. Common ADRs associated to ABZ use are mild increases in liver enzymes, and more rarely leucopenia. In agreement with published literature, mild increases in liver enzymes was the adverse event most frequently observed (17%), [2, 18, 19, 20] with return to normal levels in all cases without need to stop treatment. We recommend continuous instead of intermittent treatment since there is no evidence of increase in the frequency of ADRs and it is known that in some immunocompromised patients cyclic treatments were accompanied by cyst growth [10].

Treatment with ABZ should be started as soon as the diagnosis is suspected. In our service, even cysts bigger than 7 cm received ABZ prior to surgery, so that the patients could be operated in better conditions, allowing the use of laparoscopic procedures. In addition, long term monitoring is required to evaluate the effect of the drug before applying new treatments. In our cohort, 3 cyst progressed to regression in WHO’s classification before surgery was performed, perhaps making this procedure unnecessary. Nevertheless, surgery continues to play a central role in the management of complicated cysts (rupture, hemorrhage, compression of vital organs, etc.) and in cases where neoplastic disease is part of the differential diagnosis.

The specific serology, performed by indirect hemagglutination (IHA) (Hidatest, Lemos SRL Laboratory, Argentina) was useful when its value was positive, but their absence does not exclude the diagnosis of cystic echinococcosis. About half of the patients in our cohort had negative serology at the time of consultation. After treatment, 41% became positive, which may reflect the release of parasite antigens into the bloodstream and immune system stimulation. This indirectly confirms the parasiticidal activity of ABZ.

Although open surgery was the most used surgical technique, videoendoscopy is currently preferred due to lower complication rates, fewer hospitalization days and less morbidity.

Since environmental exposure is shared, studying the family of the index case is important to diagnose patients with early stage cysts that can most benefit from ABZ treatment. This was the case with the asymptomatic sister of a patient who was diagnosed by ultrasound and successfully received pharmacological treatment.

## Conclusions

Long term treatment with ABZ for uncomplicated cystic echinococcosis may reduce the need for hospitalization for surgery, and treatment costs.

The development of evidence based guidelines for the treatment of cystic echinococcosis is imperative, especially for children.

## Abbreviations

ABZ: Albendazole
ADR: adverse drug reaction
CT: computed tomography
IHA: indirect hemagglutination
MBZ: Mebendazole
PAIR: Puncture, aspiration, injection, and reaspiration
US: Ultrasound studies
WHO-IWGE: World Health Organization-Informal Working Group on Echinococcosis
